# DERCo: A Dataset for Human Behaviour in Reading Comprehension Using EEG

**DOI:** 10.1038/s41597-024-03915-8

**Published:** 2024-10-09

**Authors:** Boi Mai Quach, Cathal Gurrin, Graham Healy

**Affiliations:** 1 School of Computing, Dublin City University, Dublin, Ireland; 2 ML-Labs, Dublin City University, Dublin, Ireland; 3 Adapt Centre, Dublin City University, Dublin, Ireland

**Keywords:** Computational neuroscience, Scientific data

## Abstract

This paper introduces the DERCo (Dublin EEG-based Reading Experiment Corpus), a language resource combining electroencephalography (EEG) and next-word prediction data obtained from participants reading narrative texts. The dataset comprises behavioral data collected from 500 participants recruited through the Amazon Mechanical Turk online crowd-sourcing platform, along with EEG recordings from 22 healthy adult native English speakers. The online experiment was designed to examine the context-based word prediction by a large sample of participants, while the EEG-based experiment was developed to extend the validation of behavioral next-word predictability. Online participants were instructed to predict upcoming words and complete entire stories. Cloze probabilities were then calculated for each word so that this predictability measure could be used to support various analyses pertaining to semantic context effects in the EEG recordings. EEG-based analyses revealed significant differences between high and low predictable words, demonstrating one important type of potential analysis that necessitates close integration of these two datasets. This material is a valuable resource for researchers in neurolinguistics due to the word-level EEG recordings in context.

## Background & Summary

The availability of publicly accessible EEG datasets for semantic-level information in reading is limited^1^. Researchers often design and collect data with limited consideration of reuse of the data for other researchers to conduct investigations or test hypotheses, making these datasets difficult to reuse in other studies. For example, Dufau *et al*.^2^ released an EEG dataset with a thousand words to examine the time course of orthographic, lexical, and semantic influences on word-level information. Thus, it could not be reused for studies related to decision-making or reading behaviors. In contrast, Davis *et al*.^3^ conducted a reading-based experiment with complex decision-making (CDM) tasks to indicate how brain activity in CDM was distributed across frequencies and brain regions. Therefore, this dataset cannot be potentially used to explore semantic-level hypotheses.

Moreover, designing and conducting reading experiments wherein neural responses are captured is painstaking due to the complexity and time-consuming nature of successfully deploying EEG experiments. These reasons drive us to seize an opportunity to build an EEG-based reading dataset, paving the way for cutting-edge insights into cognitive processes.

The study of brain activity during reading is a complex challenge for neuroscientists^4–8^, as it involves disentangling complex cognitive processes from continuously measured brain signals that are often contaminated with various sources of non-neural activity. Three widely used measurement techniques to study brain function include functional magnetic resonance imaging (fMRI), magnetoencephalography (MEG), and electroencephalography (EEG).

Although fMRI can effectively map brain responses in different areas to linguistic stimuli at a high spatial resolution^9^, it is limited in terms of precisely measuring the timing of cognitive processes (one scan for every 1 to 3 seconds^10^). Particularly in reading, neural events often occur within milliseconds. On the other hand, MEG and EEG provide dense snapshots of brain processing at a higher temporal resolution, in the order of milliseconds range but with poorer spatial resolution than that of fMRI. Compared to EEG, MEG provides a much better balance of temporal and spatial resolution, a higher signal-to-noise ratio (SNR), and shorter procedural time than EEG^11^. However, EEG is often preferred as it is portable and less expensive, hence it has been used in a large number of neurolinguistic studies to date.

Event-related potentials (ERPs) in a continuous EEG recording are voltage fluctuations that are time-locked to particular types of events, such as the presentation time of stimuli, where averaging of the time-locked EEG signals is typically employed to improve SNR. ERPs have been extensively utilised in many language studies, especially the N400 ERP^12–18^, which typically occurs 300-500 ms after the word onset. Studies investigating the N400 have established an association with semantic complexity^17^, as an index of semantic processing. Several studies have shown that the N400 is sensitive to both linguistic and non-linguistic characteristics, including expectancy effects^12,18^, frequency^13^, orthographic neighbourhood effects^15,16^, and lexical association^14^.

Over time, EEG has become an important measurement technique not only for neuroscientific research but also for those in the field of Artificial Intelligence (AI), in particular for natural language processing (NLP). Working with EEG and AI in combination has enabled researchers to explore the similarities and differences between brain activity and AI models in language processing^19–21^, mapping neural signals to natural language^8,22^, decoding linguistic information from the brain^5,23^, or encoding language into neural activity representations^24^.

Two notable EEG-based reading datasets made available for other researchers are the Kilo-word ERP dataset and ZuCo dataset. Dufau *et al*^2^ published the Kilo-word ERP dataset in 2015, consisting of EEG data from 75 subjects performing a lexical decision task on approximately 1,000 English words. This dataset has been reused in neurolinguistic studies^25^ and for teaching ERPs^26^. However, the paradigm used to capture this data presents word stimuli independent of each other without context, i.e. word stimuli presentations are not part of a sentence. The ZuCo 1.0 dataset^27^ was first released in 2018, updated to ZuCo 2.0^28^ in 2019, and has been applied in numerous studies^8,23,29,30^. This dataset comprises EEG measures co-registered to eye-tracking during natural reading. However, the dataset’s focus is on NLP tasks such as entity recognition, relation extraction, and sentiment analysis using machine learning algorithms. Hence, the ZuCo dataset does not completely focus on single-trial responses at the word level.

To balance the disadvantages and advantages of these two datasets, our dataset focuses on single-trial neural responses collected in a narrative context. Single-trial responses in our experiment are synonymous with word-level EEG responses. This approach is particularly important as it allows us to perform analyses such as comparing high versus low predictable words. While responses may be averaged within a category (e.g. high probability), word-level (single-trial) responses are still ultimately required to perform analysis on language-related ERP components^31^.

Moreover, our dataset includes data from two types of experiments. The first one is a typical word-by-word presentation, also known as Rapid Serial Visual Presentation (RSVP), which requires participants to maintain their gaze on a location on screen where words are displayed one by one in the fovea at a fixed pace^32^. This classic RSVP ignores some properties of natural reading, namely parafoveal perception, which occurs in natural reading where when a person is fixating on a word they still perceive properties (e.g. word length) of the surrounding words in a sentence. Horizontal EOG (Electrooculargraphy) was encluded as a part of dataset, enabling researchers to examine horizontal eye movements should they wish to further investigate this. From our observation of the data, there was minimal eye movements, but users of the data may decide to impose stricter thresholds regarding minor eye movements (e.g. microsaccades). Also, this paradigm uses a fixed presentation rate for each word, effectively driving the speed at which participants read^33–35^.

A major benefit of using the RSVP word stimulus presentation approach is that it mitigates against cornea-retinal dipole activity related to eye movements that would occur during natural reading from contaminating the EEG signal recordings, which are particularly difficult to effectively remove after the fact^36^. Nevertheless, an important drawback of the RSVP approach is the reduction in visual acuity from the foveal region to the parafovea and then the periphery^37^. To overcome this, we designed the second experiment called RSVP-with-flanker paradigm. In this type of experiment, the word at fixation always reveals while the words on neighbouring sides are flanked by letter strings. Many of the studies using RSVP-with-flankers also incorporate ERPs analysis related to semantic integration^38–41^.

An important feature of this dataset is human annotation data via a behavioural word-prediction experiment conducted using Amazon Mechanical Turk^42^ for 500 participants. The word “behaviour” was intended to indicate that participants were required to predict upcoming words^43^. Behavioural experiments are designed to test a belief or prediction and to discover new insights^44^. They are frequently conducted on crowdsourcing platforms for tasks related to psychology, neuroscience, and cognition^45^. In our research, this experiment captures the predictions for next words from 500 participants, allowing us to measure the predictability of words in the context. Combining this dataset with the EEG-based reading data for 22 participants, we demonstrate ERP differences using high-and-low cloze probabilities for next-word predictions, demonstrating the complementary integration of both datasets.

We anticipate that this dataset will facilitate AI-related research, including the development of brain encoding and decoding models, research in cognitive science and linguistics, and advancing the capabilities of natural language processing systems. For instance, it will enable researchers to study the nature of brain signals underlying semantic-level information in reading contexts. Additionally, by standardising EEG data formats and providing thorough metadata annotation, this dataset will support data reuse in the co-registration of EEG with different modalities such as fMRI, MEG, eye tracking data, etc. The technical validation of this dataset, described further below, serves a proof of the quality of the datasets.

## Methods

In our experiments, we used the same materials for both the EEG-based reading and the behavioural word-prediction experiment. The script of the five articles are reported in Supplementary File 1. The study was approved by the Dublin City University Research Ethics Committee (Reference: DCUREC/2023/085) for both experiments. Participants’ responses were de-identified i.e. each individual data was represented by a unique random ID with no recording of name. They also provided permission for their data to be shared as a part of informed consent process.

### Materials

The primary purpose of this dataset was to enable researchers to investigate reading behaviours and comprehension, necessitating the selection of materials that avoided using advanced/unfamiliar words and did not require specific domain knowledge, such as that needed for understanding a scientific article. Many previous studies of brain activity during reading experiments have utilised naturalistic stories for the collection of neural data, including the Alice datasets^46^, Le Petit Prince (The Little Prince) corpus^47^, the Harry Potter datasets^48,49^, and the Adventures of Sherlock Holmes datasets^19,50^. Therefore, we found that the use of narrative stories, particularly fairy tales, fully satisfied the mentioned requirements.

Based on these considerations, five short stories from Grimms’ Fairy Tales were selected, namely: “The Mouse, the Bird, and the Sausage” (Article 1), “Straw, Coal, and Bean” (Article 2), “Poverty and Humility Lead to Heaven” (Article 3), “The Death of the Little Hen” (Article 4), and “The Wolf and the Fox” (Article 5). These fairy tales include a large, rich set of verbal descriptions without the use of advanced vocabulary, making them comprehensible to individuals with varying degrees of knowledge.

Additionally, these stories are not the commonly known, thereby mitigating the impact of familiarity. When creating the descriptive table, we removed currency symbols, numerals, and punctuation. Table 1 presents the overall descriptive statistics of the materials split by story. It shows the variability in article length, sentence structure, and word usage, with *distinct word counts* ranging from 224 to 301, and *sentence counts* from 23 to 36. The table also includes mean and standard deviation values for *words per sentence* and *word length*, indicating the average and variability within these metrics across the articles.

**Table 1:** Descriptive statistics of reading materials (M = mean, SD = standard deviation).

		Article 1	Article 2	Article 3	Article 4	Article 5
**Number of words**		564	466	493	660	776
**Number of distinct words**		243	235	226	224	301
**Number of sentences**		33	32	23	36	34
**Word per sentences**	**M**	17.09	14.56	21.43	18.33	22.82
	**SD**	8.43	7.16	7.89	13.61	11.41
**Word length**	**M**	4.17	4.09	3.89	3.83	3.97
	**SD**	1.94	2.01	1.92	1.65	1.91

### Behavioural Word-Prediction Experiment

#### Participants

A total of 500 participants completed an upcoming word prediction task (behavioural word-prediction experiment) on Mechanical Turk^42^, a crowdsourcing marketplace on Amazon. Each HIT (Human Intelligence Task) corresponded to one of the five stories, meaning that we had 100 subjects per story. Demographic information was not collected, and participants remained completely anonymous. The HITs were only available for participants in the USA, Canada, Australia, England, and Ireland, aged 18 years or older. Potential participants saw the HIT on the Amazon Mechanical Turk platform and then decided whether to complete the tasks for compensation. Before beginning the HIT, participants were required to sign a digital consent form with three conditions: 1. You voluntarily agree to participate, 2. You are 18 years of age or older, 3. You are a native English speaker (as shown in Supplementary Figure S1). Volunteers participating in our experiment were compensated with a fee of $1.2 to $1.5 per story, depending on the story’s length. Each HIT (per story) lasted 25 minutes on average.

#### Stimuli & Experimental Design

The experiment was presented to participants as an HTML webpage with the task flow controlled by jsPsych^51^, which is a JavaScript framework for creating behavioral experiments running in a web browser. The words were displayed in 21-px Arial font with a black color at the centre of the webpage, while the page had a white background color.

**Fig. 1:**
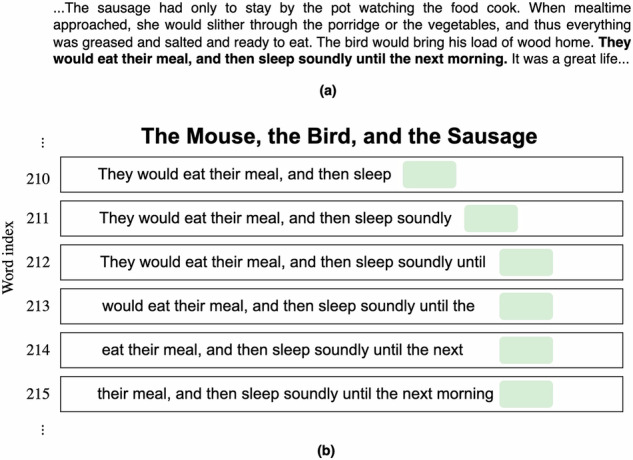
The stimulus was transcribed in article 1 for the behavioral experiment. **(a)** Transcript **(b)** A ten-word sliding window example.

**Fig. 2:**
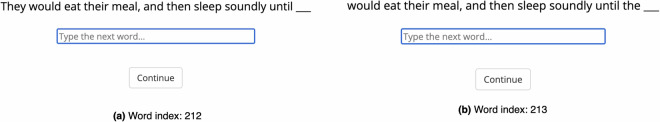
A ten-word sliding window was presented on the website for the article 1. **(a)** The next word is “the” **(b)** The next word is “next”.

This experiment is also known as cloze procedure^52^, a traditional approach which involves asking participants to predict and complete unfinished sentences or passages based on the accumulated preceding context. For each story, the title was initially displayed, and then the first two words in the content were revealed, where participants were then asked to predict (by typing) the next word. After providing their prediction, the actual upcoming word was disclosed, where participants were then asked to predict the next word in the sequence. Once ten words were displayed on the screen, the left-most word was then removed, and the upcoming word was presented. This procedure was repeated until participants provided their predictions for all upcoming missing words. The ten-word sliding window is illustrated in Fig. 1, and Fig. 2 shows the display used on the website.

Since our main focus is on next-word predictability, we derived the experimental design from papers using the cloze procedure and its application in brain activity analyses for reading and audio experiments. Goldstein *et al*.^21^ conducted a cloze experiment using a sliding-window behavioural paradigm similar to ours. In their experiment, participants initially saw two words from a story, predicted the next word, and then continued to predict subsequent words as they were revealed one by one. After displaying ten words, the earliest word was removed to make space for the next word in the sequence. Varda *et al*.^53^ implemented a similar experimental design. Additionally, they conducted a cloze experiment based on the original EEG-based reading dataset^54,55^. The materials used included a total of 205 English sentences, ranging from 5 to 15 words, with an average of 10 words. This average of ten words was also the number we chose for our experiment. Although participants had access to more context in the cloze experiment compared to the EEG-based experiment, both studies achieved significant results in analysing predictability in human brain activity based on cloze probability.

#### Procedure

Participants on Mechanical Turk^42^ found the experiment by browsing for HITs. After accepting the HIT, the participants were provided with task instructions and a web link to navigate to the experiment’s website. An informed consent form was displayed after the welcome screen. Participants then proceeded with a test trial to familiarise themselves with the experiment. Before moving on to the next step, we emphasised three important points to ensure data quality and prevent technical interruptions during the experiment: 1. Please do not skip words, 2. Please do not use online resources and, 3. Please do not reload the page. After completing the entire story (i.e. providing a complete set of upcoming word predictions), they were then asked to answer some short questions about the text they had just read to test their comprehension (included in Supplementary File 2). Finally, a survey code was provided at the end of the experiment. The participants could then use this code to complete the HIT task on the Mechanical Turk platform. A task was considered successful if the participant adhered to the rules and used the exact survey code provided by the experimenters. After experiment completion, participants were not allowed to take part in further experiments in this study.

### EEG-based Reading Experiment

#### Participants

EEG data was recorded from 22 participants who were native English speakers, originating from the UK, Ireland, and the USA. All subjects had normal or corrected-to-normal vision and no neurological disorders, by self-reporting. The participants were employees or students at Dublin City University (18-51 years of age, mean age 23.86; 7 women, 15 men). Of these, two participants (males) were excluded from data analysis due to contaminated EEG from excessive eye movements. Every participant received a voucher gratuity of €25 upon completion of the experiment, which lasted approximately two hours. All participants provided informed consent for their participation, the re-use of the data prior to the start of the data collection, and were aware that they could withdraw participation at any point during the experiment.

#### Stimuli & Experimental Design

Two types of RSVP paradigm were used for collecting the DERCo dataset for each participant: classic RSVP and RSVP-with-flanker, with a stimulus presentation rate of 200 ms. This rate is due to the fact that when a person reads a text, a word is fixated on for around 200 to 250 ms before a saccade occurs and the next word is fixated on^56,57^. Both experimental conditions were similar, except that the RSVP-with-flanker used triple-word stimuli, whereas the classic RSVP presented only one word at a time.

While the cloze procedure was suitable for the online experiment, it was not appropriate for use in the EEG experiment. For accurate time-locking in EEG analyses, it was crucial to present stimuli at a steady rate, minimising the impact of eye movements on the EEG signals i.e. using RSVP^36^. More importantly, participants in the EEG-based reading experiment were not explicitly made aware that their word predictability was being investigated. We allocated 25 minutes per story in the cloze experiment because participants needed time to type their predictions. There were five short articles, each corresponding to one session. Each session lasted approximately 4 minutes (the shortest story) to 7 minutes (the longest story).

**Fig. 3:**
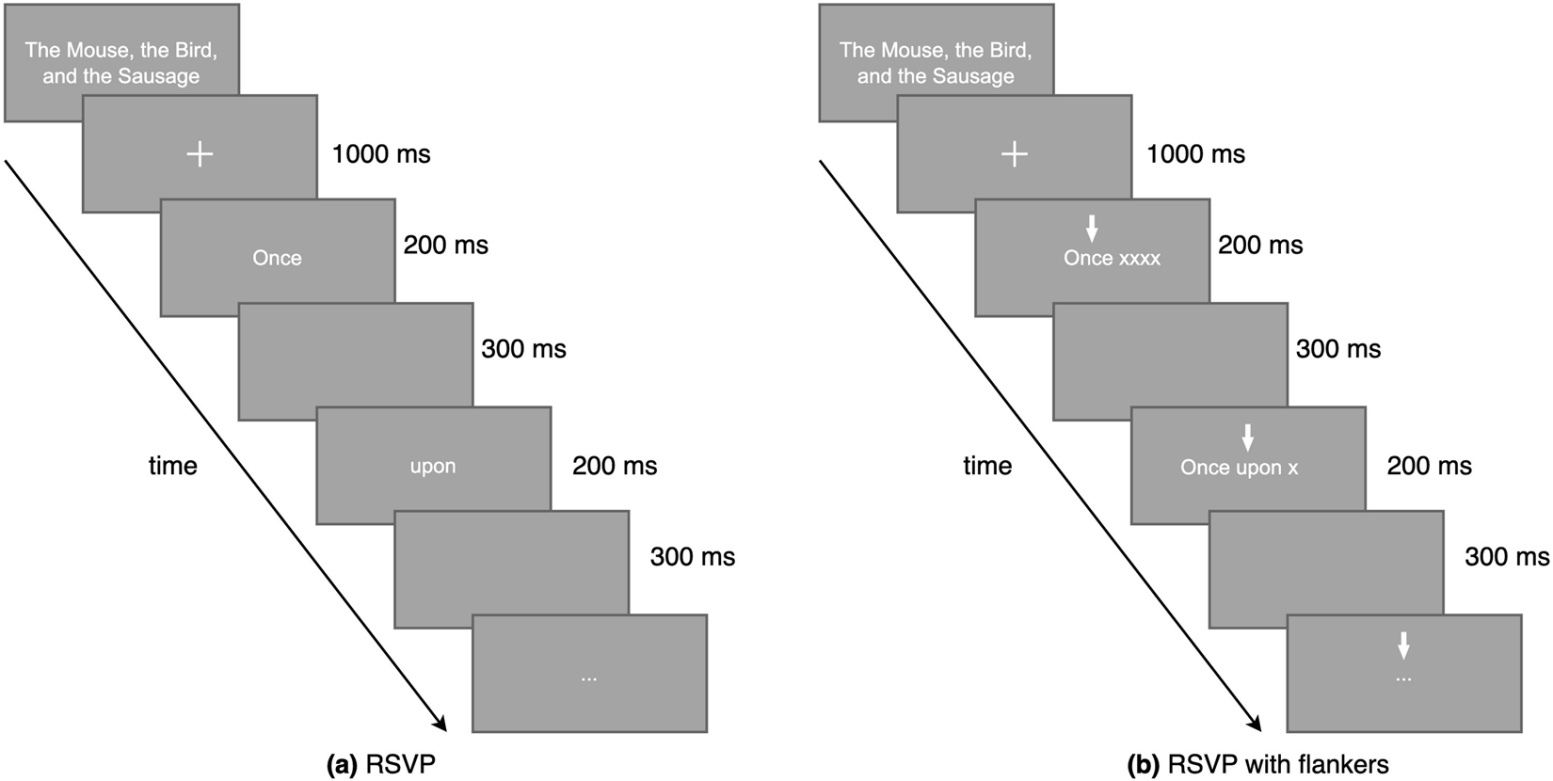
The procedure of word presentation for the first three words “Once upon a…” in **(a)** RSVP experiment **(b)** RSVP-with-flanker experiment.

In the experiment, the title was displayed at the beginning of each session. The session then began with a blank screen and a fixation cross in the centre of the screen for 1000 ms. After that, words were presented as white letters in lowercase 60-px Arial font on a gray background on a 1920 x 1080 Full HD monitor. Each stimulus word was presented for 200 ms, followed by a 300 ms blank screen. In the RSVP-with-flanker, the preceding word and the target word were revealed, while the succeeding word was hidden by a string of Xs, corresponding in length to that of the masked word. PsychoPy^58^, an open-source package for creating experiments in behavioral science, was used for stimulus presentation. The RSVP experiment implementation is illustrated in Fig. 3.

#### Procedure

The EEG data collection took place at the School of Computing in Dublin City University. After participants had been given an overview of the experiment procedure, they were provided with a Plain Language Statement outlining the study’s objectives, the attention-related metrics to be recorded, the usage of the collected experimental data, the extent of their involvement in the research study, and details about the associated risks, benefits, and inclusion criteria. Subsequently, they were required to sign a written informed consent to participate in the study.

Participants were seated in a comfortable chair, approximately 100 cm in front of a 1920 x 1080 Full HD monitor in a semi-darkened sound-attenuated room. They were instructed to read (via RSVP) the five short stories in silence, where the first three stories were presented using RSVP (i.e. word-by-word display), and the last two stories were presented using RSVP-with-flankers (i.e. three consecutive words displayed at a time with the upcoming word represented with X’s). Participants completed a test trial before conducting the real experiment. Participants were asked to remain relaxed, and to minimise their movements during RSVP presentation, a common instruction to minimize movement artefacts in EEG recordings.

The stimuli were displayed following the description in the Stimuli & Experimental Design. After the final word disappeared, the participants answered comprehension questionnaires related to what they had just read. The primary purpose of these basic questions on the story content was to ensure the subject maintained their attention during the task i.e. the participants needed to pay attention in order to be able to answer these questions afterwards.

Sessions were self-paced i.e. participants could take a 5-10 minute rest between sessions until they were ready to proceed. After completing five sessions, the participants were required to answer certain questions about their experience during the experiment. The questionnaire for the five articles are included in Supplementary File 2.

### Data acquisition

#### Behavioural Word-Prediction Dataset

We used the AWS service to save collected data from the HITs. Amazon S3^59^ was used for storage to save the raw data after participants completed the task. Each survey code corresponded to a folder for each participant, and the raw data was saved in a CSV file.

#### EEG Dataset

The EEG data was recorded using an ActiCHamp EEG system^60^ with a 32-channel active electrode cap, with electrode positions following the international 10-20 system^61^. The signals were collected at a sampling rate of 1,000 Hz with a 500 Hz IIR (Infinite Impulse Response) low-pass filter, referenced to an internal reference (virtual ground) of the amplifier. The impedance of each electrode was carefully checked before recording to ensure good conductivity, and were checked again before each session to maintain all electrode impedances below 5k*Ω*. EEG signals were continuously recorded during each session. A photodiode connected to an external circuit was affixed to the top right hand corner of the screen and was used to generate markers (corresponding with word presentations) on the EEG recording^62^. This allowed for correctly aligned epochs corresponding to word presentations to be extracted after the experiment. Participants were not able to see the visual marker used to trigger the photodiode i.e. it was physically occluded.

### Data preprocessing

#### Behavioural Word-Prediction Dataset

For each response in the prediction data, we remove punctuation and convert them to lowercase. Additionally, we also conducted checks to ensure the data quality. Namely, any spelling errors were corrected; for instance, ‘mornin’ is a typo for ‘morning’, ‘brige’ is a typo for ‘bridge’. Another scenario involves participants typing too quickly, pressing adjacent keys on their keyboards, resulting in grammatically incorrect words such as ‘sround’ instead of ‘around’ because the character ‘s’ is next to ‘a’. Importantly, these errors were not due to participants predicting incorrectly but would ultimately impact subsequent analyses. Generally, if a (misspelled) word only differed by one letter to its correct version (i.e. including a letter, removing a letter, or changing a single letter), the misspelling was rectified in the preprocessing stage.

Each response corresponded to a word at a unique location in the article. Keeping the words as indices ostensibly results in duplicates for analysis. Importantly, however, even though the words are the same, people may have different predictions or thoughts based on the context of the sentence, the topic, or their knowledge^63,64^. Thus, we combined the position and story index for each word to create new indices.

In order to organise the necessary information for use, we designed three schemas corresponding to questions and predictions. All 100 results for each story were also saved in CSV files. We have published the dataset (both raw and preprocessed data) on the Open Science Framework (OSF). Please refer to the OSF project^65^ for more information about data organisation, an introduction, size, authors, and schema of the DERCo dataset.

#### EEG Dataset

**Fig. 4:**
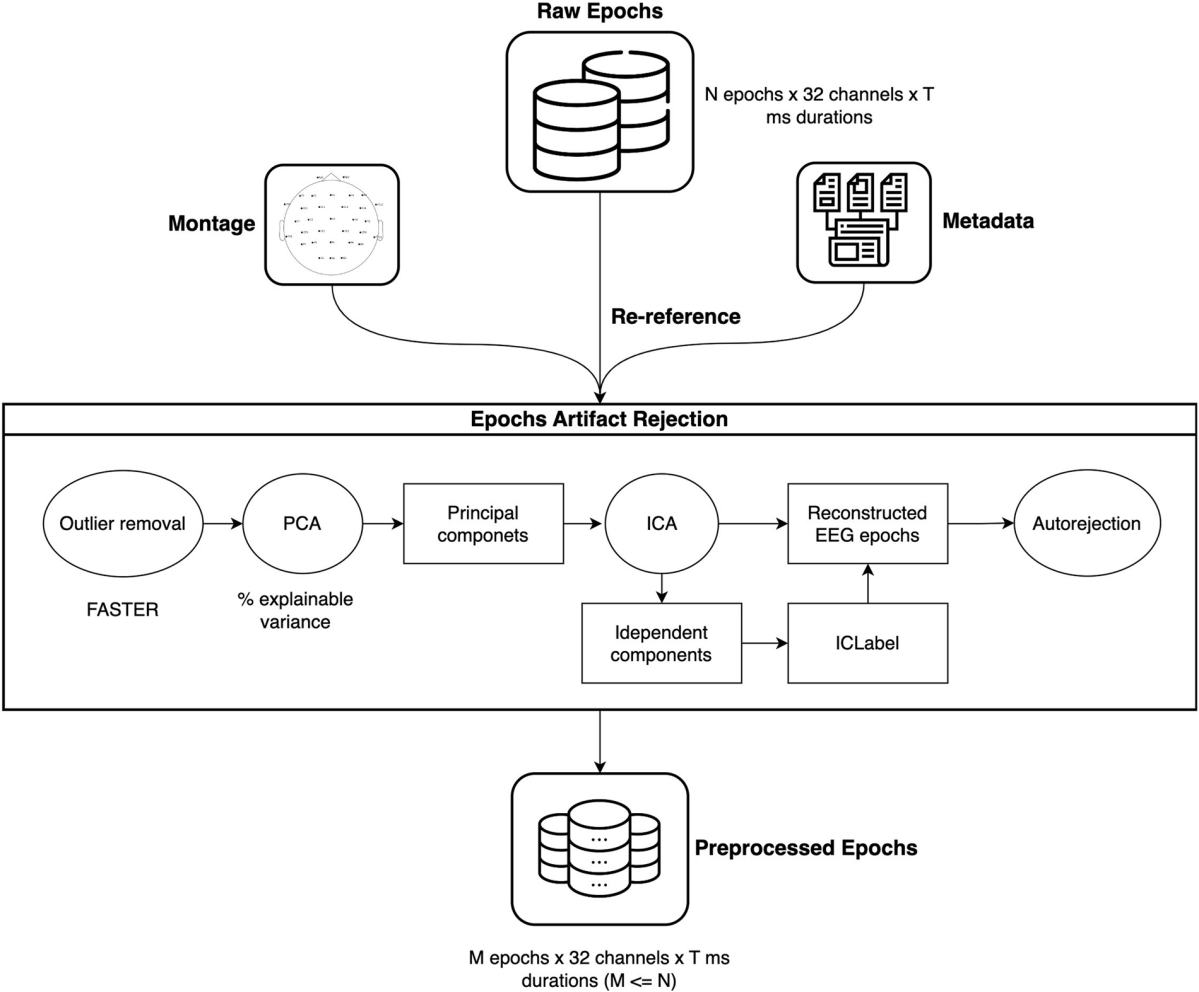
The EEG epochs data preprocessing pipeline.

The raw EEG data was filtered with bandpass cutoffs at 0.1 Hz to 45 Hz using an FIR (Finite Impulse Response) filter with a Hamming window. Subsequently, using the triggers at stimulus onsets, we epoched the raw EEG data into several segments. These raw epochs were then input into an EEG epochs data preprocessing pipeline (as described in Fig. 4) to create preprocessed epochs. The description of the preprocessing is outlined below.

**Fig. 5:**
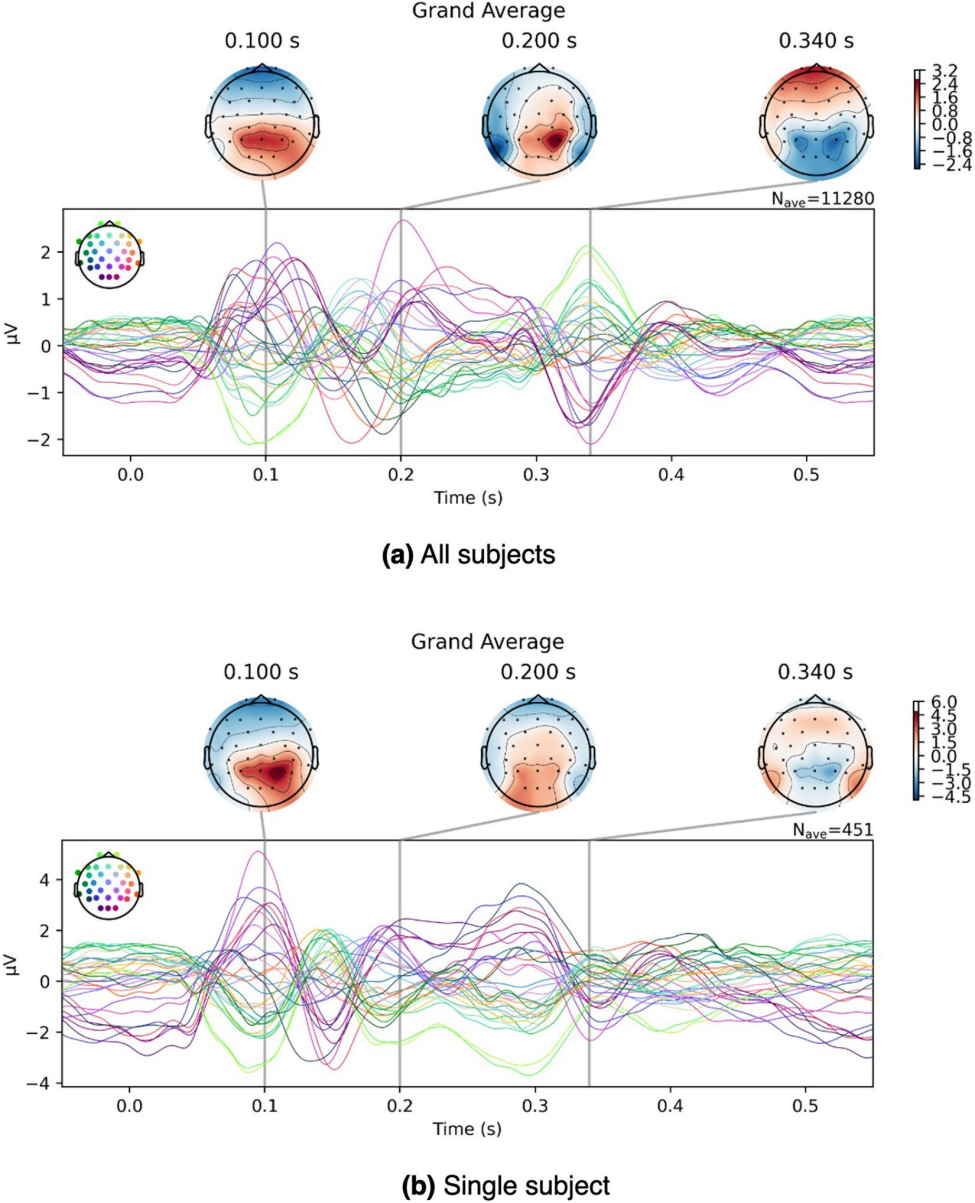
Butterfly plots for EEG averages across all words in article 1 **(a)** across subjects **(b)** single subject (JPY86). Each time-series line graph corresponds to an electrode.

**Fig. 6:**
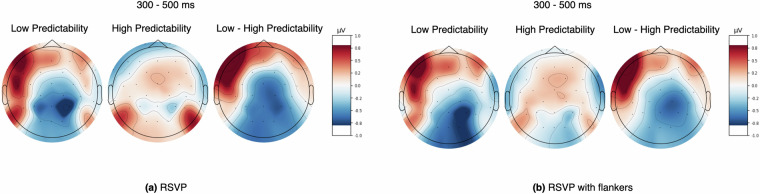
Scalp distributions of the response for low predictable words, high predictable words, and the difference between low and high predictable words (low minus high) in **(a)** RSVP experiment and **(b)** RSVP-with-flanker experiment. Amplitudes (*μV*) were averaged in 300-500 time window (for all electrodes).

**Fig. 7:**
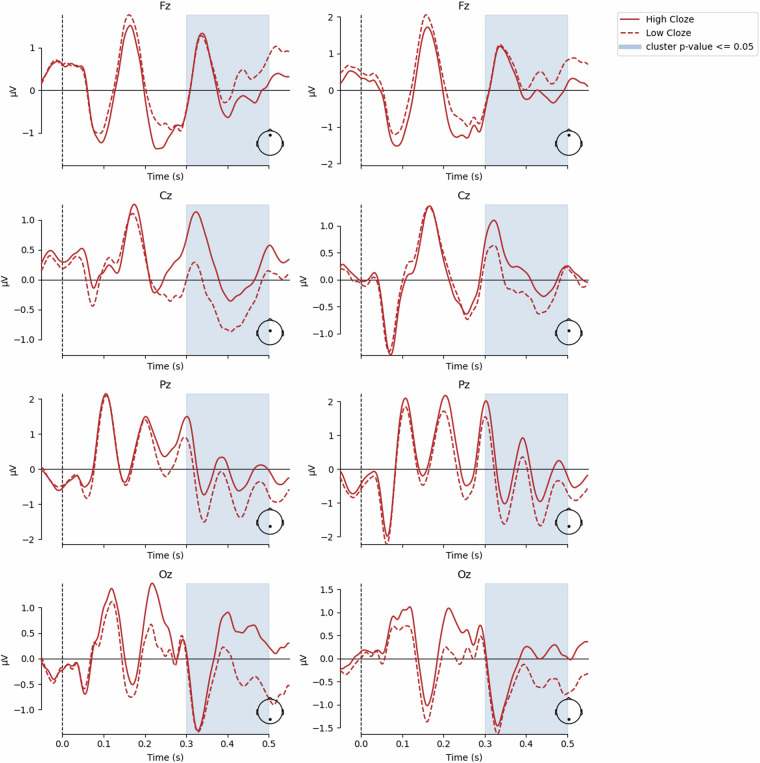
ERP activity for midline electrodes in RSVP experiment (left) and RSVP with flankers experiment (right). The time range of significant differences (*p* < 0.05) between 300ms and 500ms post-stimulus are indicated.

First of all, the raw EEG epochs were imported into the pipeline using the MNE package version 1.4^66^. Common average referencing (CAR) was applied to all data. The next stage of the pipeline aimed to mitigate artifacts in the EEG recordings. The initial step involves outlier removal using Fully Automated Statistical Thresholding for EEG artifact Rejection (FASTER)^67^. Epochs were rejected based on a global threshold on the z-score, exceeding 3 standard deviations for epoch amplitude, deviation, and variance range. The outputs were then preprocessed by applying independent component analysis (ICA)^68^ to further improve the signal-to-noise ratio (SNR). The number of principal components generated from the the pre-whitening principal component analysis (PCA) step was selected based on the percentage of explainable variance, 99% was used in this study, which was accounted for by the principal components retained after dimensionality reduction.

ICA allows the decomposition of EEG signals into independent components (ICs), and then the corrected EEG signals can be obtained by discarding ICs containing artifacts^69^. The Picard algorithm^70^ was used for maximum likelihood independent component analysis. To automatically determine which ICs contain artifacts, we applied the ICLabel classifier^71^. The remaining ICs were then reconstructed to create clean EEG epochs.

Following the ICA process, Autoreject^72^ was applied for automatically identifying and removing artifacts. Autoreject uses unsupervised learning to estimate the rejection threshold for the epochs. In order to reduce computation time, which increased with the number of epochs and channels, we fitted Autoreject on a representative subset of epochs randomly picked from 25% of the total epochs. The number of cleaned epochs ultimately was less than or equaled the number of raw epochs. Importantly, this means for some word indices in stories there are no corresponding EEG epochs after trial rejection for some participants.

## Data Records

The dataset is available on the OSF website^65^. There are two main folders corresponding to two experiments: the Behavioural Word-Prediction Experiment and the EEG-based Reading Experiment. The behavioural word-prediction experiment folder consists of three subfolders: “experienced_question”,"prediction”, and “question”. Each subfolder contains 5 CSV files corresponding to the participants’ responses across 5 articles. Supplementary File 2 contains all the questions asked in the experiment.

**Fig. 8:**
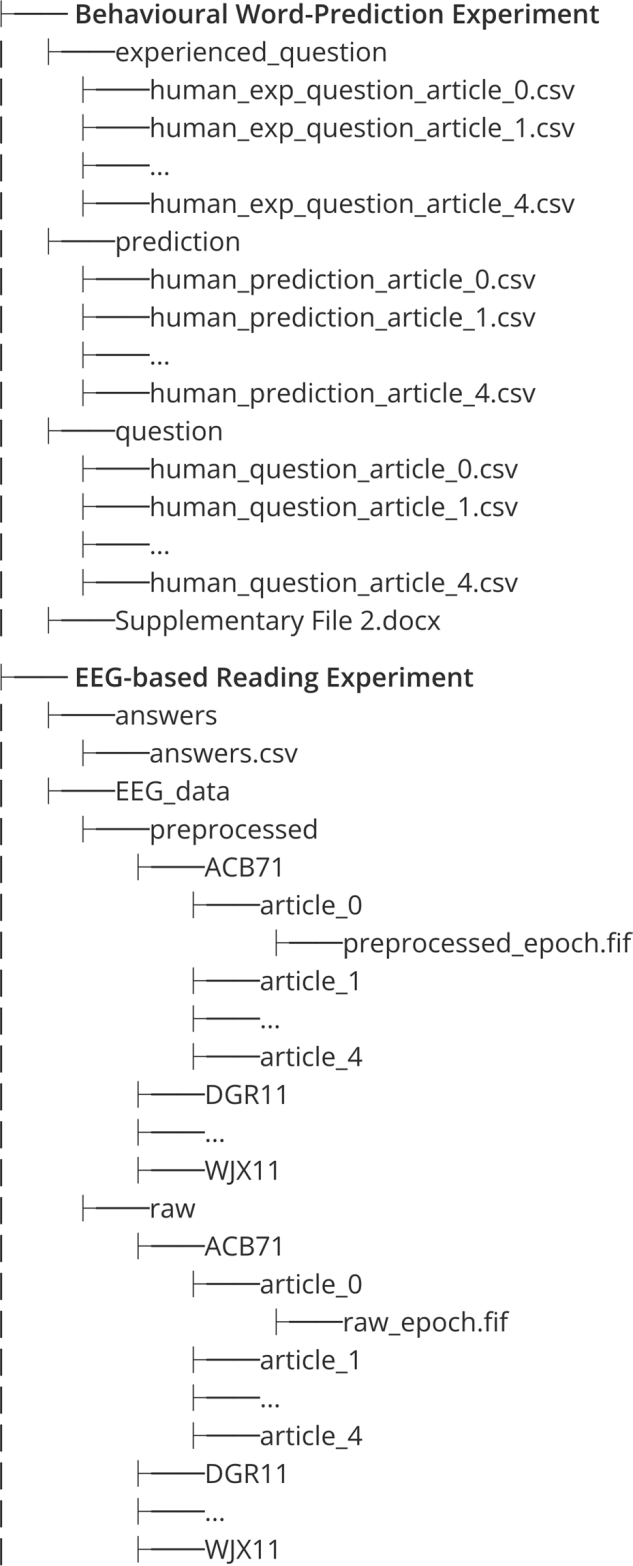
The structure of the dataset.

In the EEG-based reading experiment folder, there is an “answers” folder containing the answers of all participants collected after they completed reading each article. The main folder “EEG_data” has two subfolders: “raw” and “preprocessed”, which have the same structure. They contain 22 subfolders, each corresponding to a participant. Each participant’s folder has five subfolders for five articles, named “article_0”, “article_1”, etc. In each article folder, there is an epoch file in *fif* format. The directory tree for the repository of the DERCo project is shown in Fig. 8.

## Technical Validation

In this section, we examine next-word predictability - a key aspect in reading behaviour^73^ - to validate that the word-level predictability measures calculated in the behavioural word-prediction experiment dataset can be meaningfully integrated with the EEG-based reading experiment dataset. To achieve this, we created the DERCo dataset using data collected from two experiments: the “Behavioral Word-Prediction Experiment” and the “EEG-based Reading Experiment”. After applying the techniques described in the Data Preprocessing section, we obtained neural responses based on word-level and semantic-level information. However, we could not collect the exact predictions of the next words for each participant using the EEG-based reading experiment. Therefore, the behavioral word-prediction experiment was carried out with a large sample size to measure the predictability of each word (at a group level) in the texts. By combining the data collected from both experiments, we have EEG responses and predictability values for each word in the articles. The data in the behavioral word-prediction dataset was validated to demonstrate participants’ proficiency in predicting subsequent words and to assess participants’ reading comprehension of the articles. Given the significant results at the semantic level, we then analyse next-word predictability based on EEG signals at the word level using the predictability measures calculated from the behavioural word-prediction experiment dataset. This validation exercise is crucial to demonstrate that these two datasets are meaningfully aligned, ensuring they can be used together for effective analysis and investigation.

### Behavioural Word-Prediction Dataset

Using the *general questions* in each story (Supplementary File 2), we estimated a participant’s prior knowledge of the recently read narratives and identified whether the articles used excessively advanced or unusual vocabulary. The result indicated that a significant majority of subjects, ranging from 98% to 100% of the total, had not read the stories before. Despite the unfamiliar stories, most participants agreed that no advanced or uncommon words were used. Only the final and longest story had agreement from 89% of participants, whereas the other articles achieved percentages between 95% and 98%.

As an estimate of participants’ overall reading comprehension (as a proxy measure to ensure participants correctly read the texts), a proportion was calculated based on the number of correct answers to multiple-choice questions for each subject. As a result, a substantial number of participants, between 98 and 100 participants, showed a performance greater than or equal to 60%, indicating their ability to comprehend and recall the stories’ content to provide accurate answers after reading.

The ability to anticipate upcoming words is commonly linked with the “cloze probability” or “cloze value”^12,74,75^. It is calculated as the proportion of participants that picked the same target word for the next word prediction. Cloze values range from 0 to 1, where when 0 indicates that no participant predicted the same next word, and 1 indicating all participants predicted the same next word. Notably, high predictability was observed for all words from all parts of speech in a sentence, and not just the last words in a sentence. More than 1000 words out of about 3,000 words in all articles had a cloze probability higher than 0.5. Of these, 62.74% of words were functions words, 20.32% were nouns, 6.24% were verbs, 5.35% were adjectives and verbs, and 5.35% were classified as other. The parts of speech were identified using the Python library IPA^76^. For further details, Table 2 provides further information about top-1 accuracy for each story and word categories, including content words (nouns, verbs, adjectives, and adverbs), function words, and others. The accuracy refers to the percentage of times participants correctly predicted the upcoming words in the story. We found that the number of sentences decreases in the order of articles 4, 5, 1, 2, and 3, corresponding to a reduction in the percentages of content words in the same sequence (38.77%, 35.48%, 31.67%, 24.46%, and 19.59%). As a result, articles with more sentences show higher accuracy in predicting content words (nouns, verbs, adjectives, and adverbs). For all words, we included the descriptive statistics in Supplementary Table S1. These findings suggest that humans are proficient in anticipating next words in narrative texts when asked to do so.

**Table 2:** Human next-word prediction performance.

Story	Accuracy	No. words (p ≥ 0.5)	% nouns	% verbs	% adjs & advs	% function words	% others
1	45.02%	180	23.89%	5.00%	2.78%	60.56%	7.78%
2	40.95%	139	15.83%	2.16%	6.47%	72.66%	2.88%
3	41.34%	148	14.19%	2.70%	2.70%	75.00%	5.41%
4	55.32%	294	21.77%	7.14%	9.86%	56.80%	4.42%
5	41.60%	248	22.18%	10.48%	2.82%	58.47%	6.05%
**Total**	45.17%	1009	20.32%	6.24%	5.35%	62.74%	5.35%

% shows the percentage of correctly predicted words for each story and part of speech. “No. words (p ≥ 0.5)” shows the number of words with a cloze probability above.5.

### Neural Responses in Reading

The experimental design differed between the behavioural word-prediction experiment and the EEG-based reading experiment due to their distinct requirements, as mentioned in the procedure sections for both experiments. For the online experiment, the cloze procedure was appropriate, allowing participants to type their predictions for upcoming words. However, this method was unsuitable for the EEG-based reading experiment, which required precise time-locking of stimulus presentation to enable ERP analysis^77^. Despite the differences in the stimulus presentation paradigms between both experiments, we show significant results, demonstrating that word-level predictability measures calculated in the behavioural word-prediction experiment dataset can be meaningfully integrated with the EEG-based reading experiment dataset, as outlined below.

EEG data was segmented into epochs, starting 50 ms before and lasting 550 ms after the word-onset. Each grand-averaged word ERP was calculated by averaging the neural responses across participants for each channel generated by a given word. Figure 5 shows the EEG averages across all words for 32 electrodes in the first article between all subjects and a selected individual (user-id = JPY86). Consistent with the findings of the previous study by Dimigen *et al*.^78^, we can see a positive peak at around 100 ms post-word onset, also known as P100. A second positive peak (P200) can be identified at around 200 ms after word-onset.

For a technical validation, that incorporated both the behavioural word-prediction dataset (Mechanical Turk) with the EEG dataset, we investigated N400-like components within the 300 - 500 ms time window. The ERP was examined across midline electrodes Fz (Midline Frontal), Cz (Midline Central), Pz (Midline Parietal), and Oz (Midline Occipital). Using midline electrodes in EEG studies provides balanced recordings of brain activity. They capture significant neural activity from central brain regions involved in cognitive processes, reduce hemispheric lateralisation effects, and ensure consistency and comparability due to their standardized use across studies^79^.

We used two non-overlapping groups of *low predictable* words (cloze *p* ≤ 0.06, *n* = 989), and *high predictable* words (cloze *p* > 0.52, *n* = 962), calculated from the Mechanical Turk dataset. Figure 6 provides a more detailed view of the topographies of response differences between conditions across time, computed averages of 300 - 500 ms range in RSVP and RSVP with flankers, respectively. The color intensity indicates the mean ERP amplitude across all participants for the low and high predictability groups. As shown in Figure 6, the effect of word predictability actually modulated the ERP evoked after the word onset.

In order to capture variability in cloze values, we applied an independent samples t-test on the high and low predictability, and plotted the ERP waveforms between these conditions to examine ERP activity in the time window of 300 - 500 ms. Results from the t-test on midline electrodes and the corresponding visualisations are presented in Fig. 7. Post-hoc t-tests revealed significant differences (*p* < 0.05) between high and low predictability at all mid-line electrodes in between 300 and 500 ms post stimulus in both RSVP and RSVP-with-flankers paradigms. If conducting t-tests for all channels, approximately 70% electrodes showed significant differences (*p* < 0.05) in both experiments. In the RSVP paradigm, nine electrodes (FC1, C3, TP9, P7, TP10, T8, FT10, FC6, and F4) had no predictability effect in the 300 - 500 ms range between two conditions. The predictability effect was not found for ten channels (Fp1, FC1, C3, P7, TP10, T8, FT10, FC6, F4, and Fp2) in the RSVP-with-flanker experiment at the same time window.

These validation results show one potential analysis trajectory that necessitates the use of both the behavioural word-prediction dataset and the EEG data. Moreover, it validates that the word-level predictability measures calculated in the behavioural word-prediction experiment dataset can be meaningfully integrated with the EEG-based reading experiment dataset. Importantly, such analyses could not be performed with one dataset alone.

### Limitations

Although the EEG and behavioral word-prediction datasets provide a novel perspective on reading behaviors, some limitations still exist. Firstly, while the experimental design for EEG collection simulates a closer approximation of the natural reading scenario, it is still not completely naturalistic. However, because we focused on word-level EEG responses, a free-viewing paradigm was deemed unsuitable, and moreover, due to how eye movements generate artefacts in EEG. This is because people tend to skip easily predictable^80^ or high-frequency words^81^. Additionally, temporal overlap occurs between the ERPs during longer saccades or regressions in a sentence when using free-viewing paradigms^82^. Thus, researchers need to consider this when proposing hypotheses using this dataset.

Secondly, another limitation of the experimental setup is that instead of using two different groups of participants for the EEG experiment, one for the classic RSVP experiment and another for the RSVP-with-flankers experiment, we used the same participants for both. We used RSVP for the first three stories and RSVP-with-flankers for the remaining two, which reduced the total number of words available for analysis in each condition. This decision allowed for the EEG dataset to support analysis of the two RSVP conditions, whilst maintaining a sufficient number of participants for each.

Lastly, our EEG experiment participant pool was limited due to an unbalanced division of gender (approximately 68% male and 32% female). This gender imbalance should be remedied in future to achieve equal representation of each gender, because this gender distribution could have skewed results or prevented an effect from becoming significant due to lack of power^83^. However, our research primarily aimed to analyse next-word prediction behavior in adults aged 18 years and older, rather than focusing on gender differences. It should be acknowledged that several similar brain activity studies in reading experiments have also had unbalanced sample sizes for gender^2,27,46–48^.

## Supplementary information

Questionnaire for the five articles

Transcripts of five articles

Online Consent Form for Behavioural Word-Prediction Experiment

Human next-word prediction performance

## Data Availability

All the scripts for preprocessing can be found at https://github.com/Tayerquach/DERCo.
