# Supplementary material for: DERCo: A Dataset for Human Behaviour in Reading Comprehension Using EEG
Source: Sci Data. 2024 Oct 9;11:1104. doi: 10.1038/s41597-024-03915-8 (PMC11464549; doi:10.1038/s41597-024-03915-8)
Supplement: Supplementary file 1 — Questionnaire for the five articles [file 41597_2024_3915_MOESM1_ESM.docx]

**Supplementary File 2: Questionnaire for the five articles**

**Question about the article you have just read**

Congratulations! You have just finished the story "**The Mouse, the Bird, and the Sausage**". Please answer the following questions before moving on to the next story.

*Please respond to the following questions by placing a* ***check mark (√)***

**General questions**

1. Have you ever read this specific article before?  Yes |  No
2. Have you ever read a similar article before?  Yes |  No
3. Did the article use too many advanced/unfamiliar words?  Yes |  No
4. Estimate the percentage of content you still remember? Please indicate with a **check mark (√)**on the scale below. You can also check between markings.

| **0%** | **10%** | **20%** | **30%** | **40%** | **50%** | **60%** | **70%** | **80%** | **90%** | **100%** |
| --- | --- | --- | --- | --- | --- | --- | --- | --- | --- | --- |
|  |  |  |  |  |  |  |  |  |  |  |

**Story-related questions**

Who is responsible for bringing wood from the forest?

The Mouse

The Bird

The Sausage

The Dog

Why did the Bird refuse to go to the forest one day?

Because it was tired.

Because it wanted to cook instead.

Because other birds made fun of it.

Because mouse wants to go.

What happened to the Sausage when it went to collect wood in the forest?

It got lost.

It was attacked and eaten by a dog.

It found a treasure.

Nothing happened.

How did the Mouse try to cook after the Sausage was gone?

By grilling

By baking

By rolling itself in the pot to mix and season the food

By frying

What happened to the Bird in the end?

It was attacked by the dog

It drowned in the well

It flew away and never returned

Nothing happened

**Question about the article you have just read**

Congratulations! You have just finished the story "**Straw, Coal, and Bean**". Please answer the following questions before moving on to the next story.

*Please respond to the following questions by placing a* ***check mark (√)***

**General questions**

1. Have you ever read this specific article before?  Yes |  No
2. Have you ever read a similar article before?  Yes |  No
3. Did the article use too many advanced/unfamiliar words?  Yes |  No
4. Estimate the percentage of content you still remember? Please indicate with a **check mark (√)** on the scale below. You can also check between markings.

| **0%** | **10%** | **20%** | **30%** | **40%** | **50%** | **60%** | **70%** | **80%** | **90%** | **100%** |
| --- | --- | --- | --- | --- | --- | --- | --- | --- | --- | --- |
|  |  |  |  |  |  |  |  |  |  |  |

**Story-related questions**

How did the coal escape from the fireplace?

It rolled out by itself.

It was thrown out by the old woman.

It jumped out on its own.

It was carried out by the bean.

Why did the straw catch fire and fall into the brook?

The old woman set it on fire.

The coal accidentally ignited it.

It got wet from the water in the brook.

It burned due to the heat from the coal.

Who saved the bean after it burst from laughing?

The old woman

The tailor

The straw

The coal

How did the tailor fix the burst bean?

He glued it back together.

He used a needle and thread to sew it.

He replaced it with a new bean.

He wrapped it with a piece of cloth.

Why do all beans have a black seam since that time?

The beans were burned in the fireplace.

The old woman sewed them with black thread.

The tailor used black thread to sew the bean.

The water in the brook turned the beans black.

**Question about the article you have just read**

Congratulations! You have just finished the story " **Poverty and Humility Lead to Heaven**". Please answer the following questions before moving on to the next story.

*Please respond to the following questions by placing a* ***check mark (√)***

**General questions**

1. Have you ever read this specific article before?  Yes |  No
2. Have you ever read a similar article before?  Yes |  No
3. Did the article use too many advanced/unfamiliar words?  Yes |  No
4. Estimate the percentage of content you still remember? Please indicate with a **check mark (√)**on the scale below. You can also check between markings.

| **0%** | **10%** | **20%** | **30%** | **40%** | **50%** | **60%** | **70%** | **80%** | **90%** | **100%** |
| --- | --- | --- | --- | --- | --- | --- | --- | --- | --- | --- |
|  |  |  |  |  |  |  |  |  |  |  |

**Story-related questions**

What did the king's son do when he felt sad and full of thought?

He prayed for wealth and power.

He went to live in a cave.

He looked at the sky and sighed.

He started singing and dancing.

How did the poor gray-haired man suggest the king's son could reach heaven?

By wearing luxurious clothes and jewels.

By asking for food from compassionate hearts.

By becoming a king and ruling a kingdom.

By studying and gaining knowledge.

Why did the servants at the king's palace not believe that the beggar was the king's son?

They thought he was too well-dressed.

They didn't recognize him due to his ragged clothes.

They were told not to let anyone enter the palace.

They believed he was an imposter.

How did the ill-natured servant treat the food meant for the beggar?

He shared it with the beggar.

He kept it for himself.

He gave it to the dogs.

He threw it away.

What grew on either side of the beggar's grave after he died?

Roses on both sides.

Lilies on both sides.

Roses on one side and lilies on the other.

Nothing grew near his grave.

**Question about the article you have just read**

Congratulations! You have just finished the story " **The Death of the Little Hen**". Please answer the following questions before moving on to the next story.

*Please respond to the following questions by placing a* ***check mark (√)***

**General questions**

1. Have you ever read this specific article before?  Yes |  No
2. Have you ever read a similar article before?  Yes |  No
3. Did the article use too many advanced/unfamiliar words?  Yes |  No
4. Estimate the percentage of content you still remember? Please indicate with a **check mark (√)**on the scale below. You can also check between markings.

| **0%** | **10%** | **20%** | **30%** | **40%** | **50%** | **60%** | **70%** | **80%** | **90%** | **100%** |
| --- | --- | --- | --- | --- | --- | --- | --- | --- | --- | --- |
|  |  |  |  |  |  |  |  |  |  |  |

**Story-related questions**

What did the little hen find on Nut Mountain?

A small pebble

A large nut

A colourful feather

A shiny coin

What did the little hen want to do with the nut she found?

Share it with the little rooster

Bury it in the ground

Plant it to grow a nut tree

Eat the kernel herself

How did the little hen get into trouble with the nut kernel?

She lost it on the way down Nut Mountain

She swallowed it whole without chewing

She gave it away to the fox

She threw it into the well

Why did the little rooster need red silk from the bride?

To make a scarf for himself

To wrap around the nut kernel

To trade with the well for water

To fix the broken carriage

How did the little hen and the other animals meet their end?

They were attacked by a group of mice

They got lost in the forest

They drowned in the brook

They were turned to stone by a magical spell

**Question about the article you have just read**

Congratulations! You have just finished the story "**The Wolf and the Fox**". Please answer the following questions before moving on to the next story.

*Please respond to the following questions by placing a* ***check mark (√)***

**General questions**

1. Have you ever read this specific article before?  Yes |  No
2. Have you ever read a similar article before?  Yes |  No
3. Did the article use too many advanced/unfamiliar words?  Yes |  No
4. Estimate the percentage of content you still remember? Please indicate with a **check mark (√)**on the scale below. You can also check between markings.

| **0%** | **10%** | **20%** | **30%** | **40%** | **50%** | **60%** | **70%** | **80%** | **90%** | **100%** |
| --- | --- | --- | --- | --- | --- | --- | --- | --- | --- | --- |
|  |  |  |  |  |  |  |  |  |  |  |

**Story-related questions**

What was the main reason the fox decided to steal a lamb for the wolf?

Because the fox was feeling hungry

To please the wolf and avoid being eaten

To play a prank on the wolf

To teach the wolf a lesson about greed

How did the wolf end up getting caught while trying to steal the second lamb?

The farmer's dog attacked him

The fox betrayed him to the farmer's people

The wolf made too much noise

The sheep recognized the wolf

Why did the wolf want more pancakes after eating the first six?

He was still hungry

He wanted to share with the fox

He wanted to take some back for later

He accidentally dropped the first six

How did the farmer's wife discover the wolf when he tried to get more pancakes?

The wolf knocked over a dish

She heard the wolf howling

The fox told her about the wolf

She smelled the wolf's presence

What ultimately happened to the wolf when he tried to steal meat from the cellar?

He successfully escaped with the meat

He was caught by the farmer and killed

The fox helped him get out safely

He realized he couldn't fit through the hole and gave up
