# Supplementary material for: DERCo: A Dataset for Human Behaviour in Reading Comprehension Using EEG
Source: Sci Data. 2024 Oct 9;11:1104. doi: 10.1038/s41597-024-03915-8 (PMC11464549; doi:10.1038/s41597-024-03915-8)
Supplement: Supplementary file 2 — Transcripts of five articles [file 41597_2024_3915_MOESM2_ESM.docx]

*Supplementary File 1: Transcripts of five articles*

**The Mouse, the Bird, and the Sausage**

Once upon a time a mouse, a bird, and a sausage formed a partnership. They kept house together, and for a long time they lived in peace and prosperity, acquiring many possessions. The bird's task was to fly into the forest every day to fetch wood. The mouse carried water, made the fire, and set the table. The sausage did the cooking.

Whoever is too well off always wants to try something different! Thus one day the bird chanced to meet another bird, who boasted to him of his own situation. This bird criticized him for working so hard while the other two enjoyed themselves at home. For after the mouse had made the fire and carried the water, she could sit in the parlor and rest until it was time for her to set the table. The sausage had only to stay by the pot watching the food cook. When mealtime approached, she would slither through the porridge or the vegetables, and thus everything was greased and salted and ready to eat. The bird would bring his load of wood home. They would eat their meal, and then sleep soundly until the next morning. It was a great life.

The next day, because of his friend's advice, the bird refused to go to the forest, saying that he had been their servant long enough. He was no longer going to be a fool for them. Everyone should try a different task for a change. The mouse and the sausage argued against this, but the bird was the master, and he insisted that they give it a try. The sausage was to fetch wood, the mouse became the cook, and the bird was to carry water.

And what was the result? The sausage trudged off toward the forest; the bird made the fire; and the mouse put on the pot and waited for the sausage to return with wood for the next day. However, the sausage stayed out so long that the other two feared that something bad had happened. The bird flew off to see if he could find her. A short distance away he came upon a dog that had seized the sausage as free booty and was making off with her. The bird complained bitterly to the dog about this brazen abduction, but he claimed that he had discovered forged letters on the sausage, and that she would thus have to forfeit her life to him.

Filled with sorrow, the bird carried the wood home himself and told the mouse what he had seen and heard. They were very sad, but were determined to stay together and make the best of it. The bird set the table while the mouse prepared the food. She jumped into the pot, as the sausage had always done, in order to slither and weave in and about the vegetables and grease them, but before she reached the middle, her hair and skin were scalded off, and she perished.

When the bird wanted to eat, no cook was there. Beside himself, he threw the wood this way and that, called out, looked everywhere, but no cook was to be found. Because of his carelessness, the scattered wood caught fire, and the entire house was soon aflame. The bird rushed to fetch water, but the bucket fell into the well, carrying him with it, and he drowned.

**Straw, Coal, and Bean**

An old woman lived in a village. She had gathered a serving of beans and wanted to cook them, so she prepared a fire in her fireplace. To make it burn faster she lit it with a handful of straw. While she was pouring the beans into the pot, one of them fell unnoticed to the floor, coming to rest next to a piece of straw. Soon afterward a glowing coal jumped out of the fireplace and landed next to them.

The straw said, "Dear friends, where do you come from?"

The coal answered, "I jumped from the fireplace, to my good fortune. If I had not forced my way out, I surely would have died. I would have burned to ash."

The bean said, "I too saved my skin. If the old woman had gotten me into the pot I would have been cooked to mush without mercy, just like my comrades."

"Would my fate have been any better?" said the straw. "The old woman sent all my brothers up in fire and smoke. She grabbed sixty at once and killed them. Fortunately, I slipped through her fingers."

"What should we do now?" asked the coal.

"Because we have so fortunately escaped death," answered the bean, "I think that we should join together as comrades. To prevent some new misfortune from befalling us here, let us together make our way to another land."

This proposal pleased the other two, and they set forth all together.

They soon came to a small brook, and because there was neither a bridge nor a walkway there, they did not know how they would get across it.

Then the straw had a good idea, and said, "I will lay myself across it, and you can walk across me like on a bridge."

So the straw stretched himself from one bank to the other. The coal, who was a hot-headed fellow, stepped brashly onto the newly constructed bridge, but when he got to the middle and heard the water rushing beneath him, he took fright, stopped, and did not dare to go any further. Then the straw caught fire, broke into two pieces, and fell into the brook. The coal slid after him, hissed as he fell into the water, and gave up the ghost.

The bean who had cautiously stayed behind on the bank had to laugh at the event. He could not stop, and he laughed so fiercely that he burst. Now he too would have died, but fortunately a wandering tailor was there, resting near the brook. Having a compassionate heart, he got out a needle and thread and sewed the bean back together.

The bean thanked him most kindly. However, because he had used black thread, since that time all beans have had a black seam.

**Poverty and Humility Lead to Heaven**

There was once a king's son who went out into the world, and he was full of thought and sad. He looked at the sky, which was so beautifully pure and blue, then he sighed, and said, how well must all be with one up there in heaven. Then he saw a poor gray-haired man who was coming along the road towards him, and he spoke to him, and asked, how can I get to heaven. The man answered, by poverty and humility. Put on my ragged clothes, wander about the world for seven years, and get to know what misery is, take no money, but if you are hungry

ask compassionate hearts for a bit of bread. In this way you will reach heaven. Then the king's son took off his magnificent coat, and wore in its place the beggar's garment, went out into the wide world, and suffered great misery. He took nothing but a little food, said nothing, but prayed to the Lord to take him into his heaven. When the seven years were over, he returned to his father's palace, but no one recognized him. He said to the servants, go and tell my parents that I have come back again. But the servants did not believe it, and laughed and left him standing there. Then said he, go and tell it to my brothers that they may come down, for I should so like to see them again. The servants would not do that either, but at last one of them went, and told it to the king's children, but these did not believe it, and did not trouble themselves about it. Then he wrote a letter to his mother, and described to her all his misery, but he did not say that he was her son. So out of pity, the queen had a place under the stairs assigned to him, and food taken to him daily by two servants. But one of them was ill-natured and said, why should the beggar have the good food, and kept it for himself, or gave it to the dogs, and took the weak, emaciated beggar nothing but water. The other, however, was honest, and took the beggar what was sent to him. It was little, but he could live on it for a while, and all the time he was quite patient, but he grew continually weaker. As his illness increased, he desired to receive the last sacrament. When the mass was being celebrated, all the bells in the town and neighborhood began to ring of their own accord. After mass the priest went to the poor man under the stairs, and there he lay dead. In one hand he had a rose, in the other a lily, and beside him was a paper on which was written his history. When he was buried, a rose grew on one side of his grave, and a lily on the other.

**The Death of the Little Hen**

One time the little hen and the little rooster went to Nut Mountain, and they agreed that whoever would find a nut would share it with the other one. Now the little hen found a large, large nut, but -- wanting to eat the kernel by herself -- she said nothing about it. However, the kernel was so thick that she could not swallow it down. It got stuck in her throat, and fearing that she would choke to death, she cried out, "Little Rooster, I beg you to run as fast as you can to the well and get me some water, or else I'll choke to death."

The little rooster ran to the well as fast as he could, and said, "Well, give me some water, for the little hen is lying on Nut Mountain. She swallowed a large nut kernel and is about to choke to death on it."

The well answered, "First run to the bride, and get some red silk from her."

The little rooster ran to the bride: "Bride, give me some red silk, and I'll give the red silk to the well, and the well will give me some water, and I'll take the water to the little hen who is lying on Nut Mountain. She swallowed a large nut kernel and is about to choke to death on it."

The bride answered, "First run and get my wreath. It got caught on a willow branch."

So the little rooster ran to the willow and pulled the wreath from its branch and took it to the bride, and the bride gave him some red silk, which he took to the well, which gave him some water, and the little rooster took the water to the little hen, but when he arrived, she had already choked to death, and she lay there dead, and did not move at all.

The little rooster was so sad that he cried aloud, and all the animals came to mourn for the little hen. Six mice built a small carriage which was to carry the little hen to her grave. When the carriage was finished, they hitched themselves to it, and the little rooster drove. On the way they met the fox.

"Where are you going, little rooster?"

"I'm going to bury my little hen."

"May I ride along?"

"Yes, but you must sit at the rear, because my little horses don't like you too close to the front."

So he sat at the rear, and then the wolf, the bear, the elk, the lion, and all the animals in the forest. They rode on until they came to a brook. "How can we get across?" said the little rooster.

A straw was lying there next to the brook, and he said, "I'll lay myself across, and you can drive over me." But just as the six mice got onto the straw, it slipped into the water, and the six mice all fell in and drowned.

They did not know what to do, until a coal came and said, "I am large enough. I will lay myself across and you can drive over me." So the coal laid itself across the water, but unfortunately it touched the water, hissed, and went out; and it was dead.

A stone saw this happen, and wanting to help the little rooster, it laid itself across the water. The little rooster pulled the carriage himself. He nearly reached the other side with the dead little hen, but there were too many others seated on the back of the carriage, and the carriage rolled back, and they all fell into the water and drowned.

Now the little rooster was all alone with the dead little hen. He dug a grave for her and laid her inside. Then he made a mound on top, and sat on it, and grieved there so long that he too died. And then everyone was dead.

**The Wolf and the Fox**

A wolf and a fox once lived together. The fox, who was the weaker of the two, had to do all the hard work, which made him anxious to leave his companion. One day, passing through a wood, the wolf said, “Red-fox, get me something to eat, or I shall eat you.” The fox answered, “I know a place where there are a couple of nice young lambs; if you like, we will go and fetch one.” This pleased the wolf, so they went. The fox stole one, brought it to the wolf, and then ran away, leaving his comrade to devour it. This done, the wolf was not content, but wishing for the other, went himself to fetch it; and being very awkward, the old sheep saw him, and began to cry and bleat so horribly that the farmer’s people came running to see what was the matter. Of course they found the wolf there, and beat him so unmercifully, that, howling and limping, he returned to the fox. “You had already shown me how, so I went to fetch the other lamb,” said he, “but the farmer’s people discovered me, and have nearly killed me.” “Why are you such a glutton?” replied the fox. The next day they went again into the fields. “Red-fox,” said the wolf, “get me something quickly to eat, or I shall eat you!” “Well,” replied the fox, “I know a farm, where the woman is baking pancakes this evening; let us go and fetch some.” They went accordingly, and the fox, slipping round the house, peeped and sniffed so long, that he found out at last where the dish stood, then quietly abstracting six pancakes, he carried them to the wolf. “Here is something for you to eat,” said he, and then went away. The wolf had swallowed the six pancakes in a very short space of time, and said, “I should very much like some more.” But going to help himself, he pulled the dish down from the shelf; it broke into a thousand pieces, and the noise, in addition, brought out the farmer’s wife to discover what was the matter. Upon seeing the wolf, she raised such an alarm, that all the people came with sticks or any weapon they could snatch. The consequence was that the wolf barely escaped with his life; he was beaten so severely that he could scarcely hobble to the wood where the fox was. “Pretty mischief you have led me into,” said the wolf, when he saw him, “the peasants have caught, and nearly flayed me.” “Why, then, are you such a glutton?” replied the fox. Upon a third occasion, being out together, and the wolf only able with difficulty to limp about, he nevertheless said again, “Red-fox, get me something to eat, or I shall eat you!” “Well,” said the fox, “I know a man who has been butchering, and has all the meat salted down in a tub in his cellar. We will go and fetch it.” “That will do,” said the wolf, “but I must go with you, and you can help me to get off, if anything should happen.” The fox then showed him all the by-ways, and at last they came to the cellar, where they found meat in abundance, which the wolf instantly greedily attacked, saying at the same time to himself, “Here, there is no occasion to hurry.” The fox also showed no hesitation, only, while eating, he looked sharply about him, and ran occasionally to the hole by which they had entered in order to try if he was still small enough to get out by the same way he had come in. “Friend fox,” said the wolf, “pray tell me why you are so fidgety, and why you run about in such an odd manner.” “I am looking out, lest anyone should come,” replied the cunning creature. “Come, are you not eating too much?” “I am not going away,” said the wolf, “until the tub is empty; that would be foolish!” In the meantime, the farmer, who had heard the fox running about, came into the cellar to see what was stirring, and upon the first sight of him, the fox with one leap was through the hole and on his way to the wood. But when the wolf attempted to follow, he had so increased his size by his greediness, that he could not succeed, and stuck in the hole, which enabled the farmer to kill him with his cudgel. The fox, however, reached the wood in safety, and rejoiced

to be freed from the old glutton.
