# Supplementary figures and images for: DERCo: A Dataset for Human Behaviour in Reading Comprehension Using EEG
Source: Sci Data. 2024 Oct 9;11:1104. doi: 10.1038/s41597-024-03915-8 (PMC11464549; doi:10.1038/s41597-024-03915-8)

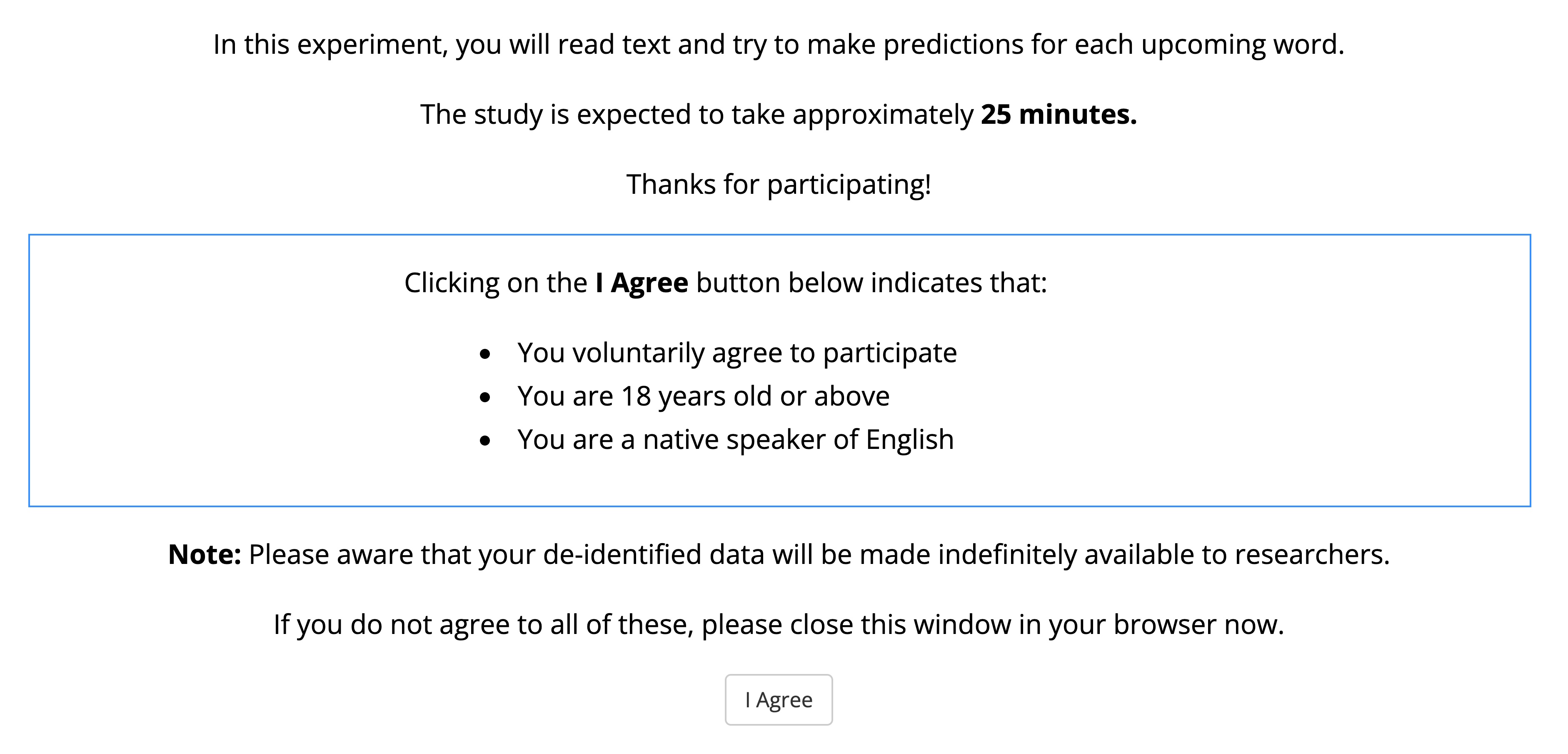

Supplement: Supplementary file 3 — Online Consent Form for Behavioural Word-Prediction Experiment [file 41597_2024_3915_MOESM3_ESM.jpg]
